# Comparison of the Clinical Characteristics and Outcome of Benign and Malignant Eyelid Tumors: An Analysis of 4521 Eyelid Tumors in a Tertiary Medical Center

**DOI:** 10.1155/2015/453091

**Published:** 2015-11-08

**Authors:** Yu-Yun Huang, Wen-Yih Liang, Chieh-Chih Tsai, Shu-Ching Kao, Wei-Kuang Yu, Hui-Chuan Kau, Catherine Jui-Ling Liu

**Affiliations:** ^1^Department of Ophthalmology, Taipei Veterans General Hospital, No. 201, Sec.2, Shih-Pai Road, Taipei 112, Taiwan; ^2^School of Medicine, National Yang-Ming University, Taipei, Taiwan; ^3^Department of Pathology and Laboratory Medicine, Taipei Veterans General Hospital, Taiwan; ^4^Department of Ophthalmology, Koo Foundation Sun Yat-Sen Cancer Center, Taipei 112, Taiwan

## Abstract

We retrospectively reviewed the clinical features and outcome of benign and malignant eyelid tumors from 1995 to 2015 in a tertiary medical center. Among 4,521 histologically confirmed eyelid tumors, 4,294 (95.0%) were benign tumors and 227 (5.0%) were malignant tumors. The mean age at diagnosis was significantly higher in patients with malignant lid tumors than those with benign lid tumors (72.5 and 55.4 years, resp., *p* < 0.001). The most common benign eyelid tumors were intradermal nevus (21.1%), followed by seborrheic keratosis (12.6%) and xanthelasma (11.2%). The most common malignant eyelid tumors were basal cell carcinomas (57.8%), followed by sebaceous gland carcinomas (21.1%) and squamous cell carcinomas (10.1%). There was a relative male predominance (63.4% and 49.2%, resp., *p* < 0.001) and higher recurrence rate (11.9% and 4.4%, resp., *p* < 0.001) in malignant lid tumors as compared with those of benign lid tumors. Twenty-two patients (9.7%) received orbital exenteration/enucleation. Eight patients (3.5%) with malignant lid tumors died of disease. Patients with eyelid melanoma were associated with a high mortality rate (25.0%). It is important to differentiate between benign and malignant eyelid tumors, because they may cause cosmetic disfigurement and severe morbidity, especially in those with malignant eyelid tumors.

## 1. Introduction

Eyelid skin is not only the thinnest skin of the body but also among the most common sunlight-exposed areas of skins. Apart from subcutaneous fat layer, eyelid contains all other skin structures that can be the origin of various benign or malignant tumors. Eyelid tumors could be cosmetically disturbing to patients as well as diagnostically difficult for family physicians, dermatologists, and ophthalmologists. Although there are some studies of eyelid tumors in the literature, most of them focused on the relative frequency of benign and malignant eyelid tumors [1–4]. This is the first study to investigate and compare the clinical features and outcome between benign and malignant eyelid tumors.

## 2. Materials and Methods

We retrospectively reviewed the medical records of all patients with histologically confirmed eyelid tumors who were treated at Taipei Veterans General Hospital between January 1995 and July 2015. Collected data included age, gender, location and size of the tumor, recurrence, and especially the treatment and outcome of malignant eyelid tumors. The clinical characteristics and outcome difference between benign and malignant eyelid tumors were compared.

### 2.1. Statistical Analysis

SPSS computer statistical software (version 20.0; SPSS, Chicago, USA) was used for statistical analysis. Significant differences between two groups were studied using 2-tailed Fisher's exact test and Mann-Whitney test. A value of *p* < 0.05 was considered statistically significant.

## 3. Result

During the 20-year interval, a total of 4,521 eyelid lesions from 4,243 patients with histopathologic confirmation were included in our study, with 4,294 (95.0%) benign tumors and 227 (5.0%) malignant tumors. The demographic data and clinical features of benign eyelid tumors are shown in Table 1. In the group of benign lesions, the most common diagnoses were intradermal nevus (21.1%), seborrheic keratosis (12.6%), xanthelasma (11.2%), and epidermal cysts (8.2%). The demographic data, treatment, and outcome of malignant eyelid tumors are summarized in Table 2. In the group of malignant tumors, the most common tumors were basal cell carcinoma (BCC, 57.8%), which were predominantly found on the lower eyelids (78.3%) and in male patients (68.7%). Sebaceous gland carcinoma (SGC) was the second most common eyelid malignancy (21.1%) and showed a predilection for the upper eyelid involvement (59.6%) and female predominance (58.3%). Squamous cell carcinoma (SCC) was the third most common eyelid malignancy (10.1%) and showed a male predominance (69.6%). The other common eyelid malignancy is melanoma (3.5%).  Table 3 shows the comparison of clinical characteristics between benign and malignant eyelid tumors. There was a relative male predominance among patients with malignant eyelid tumors as compared with those of benign eyelid tumors (63.4% and 49.2%, resp., *p* < 0.001). In addition, malignant lid tumors tended to present as an ill-defined lesion (89.2% and 7.6%, resp., *p* < 0.001) and locate in the lower eyelid (59.4% and 42.9%, resp., *p* < 0.001), as compared with those in benign lid tumors. Most patients with malignant lid tumors required wide excision and reconstructive surgery. Fifteen patients (6.6%) underwent adjunct therapy including chemotherapy, radiotherapy, or combined chemoradiotherapy. Twenty-two patients (9.7%) received advanced orbital exenteration or enucleation. The tumor recurrence was significantly higher in patients with malignant eyelid tumors as compared with those with benign lid tumors (11.9% and 4.4%, resp., *p* < 0.001). Eight patients (3.5%) with malignant lid tumors died of disease. Among these, patients with eyelid melanoma were associated with a high mortality rate (25.0%).

## 4. Discussion

The most important function of eyelids is designed to protect the eyeball. Despite their small surface area, they are among the most sunlight-exposed area of skins. The thin skin of the eyelids is particularly sensitive to various irritants and UV and is thus prone to develop eyelid tumors. Approximately 5 to 10 percent of all skin cancers occur on the eyelids [5]. We demonstrated in this study that malignant eyelid tumors showed some distinctive demographic and clinical features and treatment outcome as compared to benign lid tumors.

Most benign eyelid tumors in the present study occurred in relatively young individuals as compared to malignant lid tumors, which was similar to other studies [2, 4]. Apart from SGC and infiltrated adenoid cystic carcinoma, most malignant eyelid tumors in current study showed a strong male predominance (Table 2). However, there was no significant sex distinction among patients with benign eyelid tumors. In addition, benign eyelid tumors occurred more commonly in the upper eyelid. Conversely, malignant lid tumors tended to locate in the lower eyelid, which could be attributed to most BCC with a predilection for the lower eyelid. In contrast to other malignant lid tumors, SGC often originated in the upper eyelid (Table 2), reflecting the greater numbers of meibomian glands and glands of Zeis there. On the other hand, SGC occurred more frequently in females. In the literature, a higher incidence of SGC for females is also suggested with a female to male ratio of 1.5 to 1.0 [6–11]. The incidence of the malignant eyelid tumors is subject to geographical variation. Genetics and racial predisposition may also play a role [9]. BCC is the most common eyelid malignancy worldwide and accounts for 80–95% of all eyelid malignancy [5, 12]. However, in various Chinese studies, the percentage of BCC in all eyelid malignancy was not so much [4, 9, 13, 14]. In our study, BCC remained the most frequently malignant eyelid tumors (57.8%). SGC is very rare in Western literature. However, SGC was the second most common malignant eyelid tumor in our series and accounted for 21.1%, which was similar to some studies in Asia [9, 13–16].

In our series, malignant eyelid tumors often presented as an ill-defined lesion and usually required wide excision and reconstructive surgery that may cause cosmetic disfigurement and severe morbidity. Twenty-two patients (9.7%) required advanced orbital exenteration or enucleation in our study. Tumor recurrence developed in about 11.9% of cases of malignant eyelid tumors. Apart from infiltrated adenoid cystic carcinoma from lacrimal gland, most tumor recurrence developed in patients with SCC (30.4%) and SGC (20.8%). Although SCC and SGC are less common than BCC, they may behave biologically more aggressive and are potentially lethal tumors. The mortality rate of malignant lid tumors in current study was 3.5%, and SCC and melanoma were the most common cause of death (Table 2). Particularly, eyelid malignant melanoma remained the most high mortality rate (25%) in our studies. Treatment for cutaneous melanoma often requires wide surgical excision with histologic assurance of complete tumor removal. Till now, there is no generally accepted consensus regarding the appropriate surgical margins for eyelid melanoma. Safety margins for other cutaneous melanomas cannot be applied in eyelid melanoma for anatomic and functional reasons, because such recommendations may lead to severe ocular complications, even loss of the globe and vision. Mouriaux et al. suggest a “slow-Mohs” micrographic surgery for eyelid melanomas in order to preserve ocular integrity [17].

## 5. Conclusion

Although benign lesions comprise the majority of eyelid tumors, it is important to differentiate between benign and malignant eyelid tumors by their demographic and clinical characteristics. Early detection and appropriate treatment can reduce the risk of cosmetic disfigurement and morbidity induced by these tumors.

## Figures and Tables

**Table 1:** The demographic data and clinical features of benign eyelid tumors.

Benign eyelid tumors (*n* = 4294)	Number	(%)	Gender (M/F)	Mean age	(years, range)	Laterality^*∗*^ (R/L/B)	Location^*∗*^ (U/L/B)
Intradermal nevus	905	(21.1%)	296/609	46.0	(8–95)	425/420/9	350/423/10
Seborrheic keratosis	540	(12.6%)	348/192	66.9	(21–93)	230/262/18	175/274/6
Xanthelasma	483	(11.2%)	144/339	52.4	(3–87)	199/217/46	245/172/8
Epidermal cyst	350	(8.2%)	237/113	59.6	(0.5–94)	150/167/9	200/74/4
Chronic inflammation	321	(7.5%)	169/152	55.3	(1–91)	134/150/9	126/128/1
Verruca vulgaris	255	(5.9%)	162/93	63.0	(8–93)	101/130/5	125/80/1
Squamous papilloma	147	(3.4%)	92/55	62.3	(6–100)	51/80/4	74/36/2
Fibrosis with granulation	115	(2.7%)	61/54	46.5	(1–90)	55/55/2	58/39/0
Skin tag	101	(2.4%)	63/38	63.1	(17–90)	44/42/3	52/34/0
Papilloma cutis	95	(2.2%)	51/44	60.3	(10–94)	50/44/1	53/32/4
Chalazion	80	(1.9%)	35/45	43.6	(1–93)	37/39/4	37/45/3
Benign keratosis	71	(1.7%)	40/31	62.0	(13–90)	33/37/1	37/25/0
Keratinous cyst	62	(1.4%)	31/31	60.1	(16–90)	23/32/3	33/17/2
Compound nevus	51	(1.2%)	20/31	27.6	(6–60)	23/24/0	20/27/1
Sebaceous gland hyperplasia	51	(1.2%)	27/24	58.3	(15–92)	20/27/1	24/21/0
Hemangioma	46	(1.1%)	22/24	48.2	(3–81)	21/24/0	24/19/0
Milia	38	(0.9%)	20/18	62.9	(17–91)	11/18/4	16/11/0
Lipoma	24	(0.6%)	13/11	62.2	(32–81)	8/7/4	7/10/0
Pilomatrixoma	23	(0.5%)	12/11	34.2	(2–82)	11/11/0	16/5/0
Bowen disease	5	(0.1%)	3/2	71.3	(61–84)	3/1/0	3/1/0
Others^*∗∗*^	531	(12.4%)	258/273	57.1	(1–95)	247/256/19	259/224/3

^*∗*^Total number not equal to 100% of cases because of incomplete chart information or data missing.

^*∗∗*^Others including neurofibroma, lentigo simplex, solar elastosis, calcinosis cutis, junctional nevus, trichilemmal cyst, trichoepithelioma, pyogenic granuloma, freckle, and molluscum contagiosum.

**Table 2:** The demographic data, clinical features, treatment, and outcome of malignant eyelid tumors.

Malignant eyelid tumors (*n* = 227)	Number (%)	Gender (M/F)	Mean age	(years, range)	Laterality^*∗*^ (R/L/B)	Location^*∗*^ (U/L/B)	Adjunct treatment		Recurrence	Mortality
							Chemo/radiation/CCRT	Exenteration/enucleation	Number (%)	Number (%)
Basal cell carcinoma	131 (57.8%)	90/41	72.5	(21–93)	55/64/3	25/90/0	0/0/0	1/0	8 (6.1%)	0 (0%)
Sebaceous gland carcinoma	48 (21.1%)	20/28	74.1	(44–91)	23/25/0	27/19/1	0/3/0	5/0	10 (20.8%)	1 (2.1%)
Squamous cell carcinoma	23 (10.1%)	16/7	71.7	(40–93)	8/15/0	7/6/0	0/0/4	2/2	7 (30.4%)	3 (13.0%)
Melanoma	8 (3.5%)	6/2	67.4	(21–84)	2/6/0	3/2/0	1/0/0	5/0	0 (0%)	2 (25.0%)
Adenocarcinoma	5 (2.2%)	4/1	73.8	(64–88)	1/4/0	0/1/0	0/1/1	3/0	0 (0%)	1 (20%)
Adenoid cystic carcinoma	2 (0.9%)	0/2	53.0	(47–59)	1/1/0	1/0/0	0/2/0	2/0	1 (50%)	0 (0%)
Others^*∗∗*^	10 (4.4%)	8/2	71.7	(46–90)	5/4/0	5/1/0	1/1/1	2/0	1 (10%)	1 (10%)

^*∗*^Total number not equal to 100% of cases because of incomplete chart information or data missing.

^*∗∗*^Others including small cell carcinoma, epidermoid carcinoma, merkel cell carcinoma, B-cell lymphoma, T-cell lymphoma, lymphoepithelial carcinoma, invasive nonkeratinizing carcinoma, myeloid sarcoma, angiosarcoma, and malignant proliferating pilar tumor.

**Table 3:** Comparison of demographic, clinical characteristics, and recurrence between benign eyelid tumors and malignant eyelid tumors.

Type of tumor	Benign eyelid tumors (*n* = 4294)	Malignant eyelid tumors (*n* = 227)	*p* value
	Number (%)	Number (%)	
Gender^*∗*^			
Male	1976 (49.2)	144 (63.4)	<0.001
Female	2040 (50.8)	83 (36.6)	
Mean age (years, range)	55.4 ± 20.0 (0.5–100)	72.5 ± 12.8 (21–93)	<0.001
Location^*∗∗*^			
Upper only	2025 (55.9)	83 (40.1)	<0.001 (U : L)
Lower only	1558 (42.9)	123 (59.4)	
Both	45 (1.2)	1 (0.5)	
Laterality^*∗∗*^			
Right only	1804 (44.7)	95 (43.8)	0.33 (R : L)
Left only	1976 (48.9)	119 (54.8)	
Bilateral	258 (6.4)	3 (1.4)	
Recurrence	189 (4.4)	25 (11.0)	<0.001

^*∗*^278 patients of benign eyelid tumors had at least two histopathologic diagnoses because of 2 or more lesions in the eyelids, with 4294 lesions among 4016 patients of benign eyelid tumors.

^*∗∗*^Total number not equal to 100% of cases because of incomplete chart information or data missing.
